# Lipomatous metaplasia in cardiac CT: when ‘normal’ extracellular volume does not indicate myocardial viability - a case report

**DOI:** 10.1093/ehjcr/ytaf612

**Published:** 2025-12-01

**Authors:** Konrad Pieszko, Clara Rodríguez González, Rubén Leta Petracca, David Viladés Medel

**Affiliations:** Department of Cardiology, Hospital de la Santa Creu I Sant Pau, Carrer de Sant Quintí, 89, Horta-Guinardó, Barcelona 08025 Barcelona, Spain; Department of Interventional Cardiology and Cardiac Surgery, University of Zielona Góra, ul. Zyty 28, 65-046 Zielona Góra, Poland; IR-Hospital Santa Creu I Sant Pau, IIB-Sant Pau, Carrer de Sant Quintí, 77, Horta-Guinardó, Barcelona 08025 Barcelona, Spain; Department of Cardiology, Hospital de la Santa Creu I Sant Pau, Carrer de Sant Quintí, 89, Horta-Guinardó, Barcelona 08025 Barcelona, Spain; Department of Cardiology, Hospital de la Santa Creu I Sant Pau, Carrer de Sant Quintí, 89, Horta-Guinardó, Barcelona 08025 Barcelona, Spain; IR-Hospital Santa Creu I Sant Pau, IIB-Sant Pau, Carrer de Sant Quintí, 77, Horta-Guinardó, Barcelona 08025 Barcelona, Spain; Department of Cardiology, Hospital de la Santa Creu I Sant Pau, Carrer de Sant Quintí, 89, Horta-Guinardó, Barcelona 08025 Barcelona, Spain; IR-Hospital Santa Creu I Sant Pau, IIB-Sant Pau, Carrer de Sant Quintí, 77, Horta-Guinardó, Barcelona 08025 Barcelona, Spain; Centro de Investigaciones en Red en Enfermedades Cardiovasculares, CIBER-CV, Instituto Carlos III, Av. Monforte de Lemos, 3-5. Pabellón 11. Planta 0 28029 Madrid, Spain

**Keywords:** Dual Energy CT, Cardiac Magnetic Resonance, Multimodal Imaging, Lipomatous Metaplasia, Coronary Artery Disease, Case Report

## Abstract

**Background:**

Extracellular-volume (ECV) mapping derived from dual-energy cardiac computed tomography (DE-CT) demonstrates potential for informing myocardial-viability assessment. However, intramyocardial lipomatous metaplasia (LM)—a frequent sequel of chronic infarction—contains little or no interstitial space and may therefore normalize ECV values, falsely suggesting viable tissue.

**Case Summary:**

A 65-year-old man with chronic total occlusion of the left anterior descending (LAD) artery underwent multimodality imaging to guide revascularisation. Cardiac magnetic resonance (CMR) demonstrated a transmural, thinned scar in the antero-septal apex and sub-endocardial fibrosis in basal–mid anterior segments. DE-CT demonstrated low attenuation in the same territory, whereas ECV mapping revealed normal values in the scarred apex (comparable to non-infarcted myocardium) and mildly elevated ECV in the basal–mid anterior segments with non-transmural scar. The presence of chemical-shift artefacts on cine CMR, along with further attenuation reduction on DE-CT, confirmed intramyocardial fat, explaining the paradoxically low ECV in this region. Ultimately, the integrated assessment of limited viability, extensive scar remodelling, and high-risk procedural features led to the decision to defer revascularization.

**Discussion:**

LM can obscure chronic infarcted myocardium on CT-derived ECV maps by abolishing extracellular expansion and generating deceptively normal values. Reliable interpretation mandates correlation with tissue attenuation, mono-energetic reconstructions, or fat-sensitive CMR sequences. DE-CT affords single-acquisition, high-resolution characterization of both ECV and tissue composition, but its quantitative outputs must be contextualized to avoid misclassification of viability. Awareness of this pitfall is essential for accurate decision-making in chronic ischaemic heart disease and underscores the continuing need for integrated multimodality imaging.

Learning points

- Myocardial scars with fat tissue infiltration (lipomatous metaplasia) can have normal extracellular volume, therefore it must be interpreted in the context of signal intensity in the region of interest
- Dual energy computed tomography allows for extracellular volume calculation from single acquisition, thus making it a potentially valuable tool in viability assessment.

## Introduction

A 65-year-old male with chronic coronary artery disease and a history of ischaemic stroke was admitted for a multimodal imaging evaluation to guide decisions regarding potential revascularization. He presented with grade II stable angina and overall good functional capacity, with no clinical history of acute myocardial infarction (MI). An invasive coronary angiography (ICA) performed seven months earlier revealed chronic total occlusions (CTOs) of both the proximal left anterior descending (LAD) artery and the posterior descending artery (PDA). The patient had multiple risk factors, including hypertension, diabetes, and hyperlipidaemia. Despite medical therapy, his lipid profile remained suboptimal, with a low-density lipoprotein level of 72 mg/dL and a markedly elevated lipoprotein(a) level of 220 nmol/L.

## Summary figure

**Figure:**
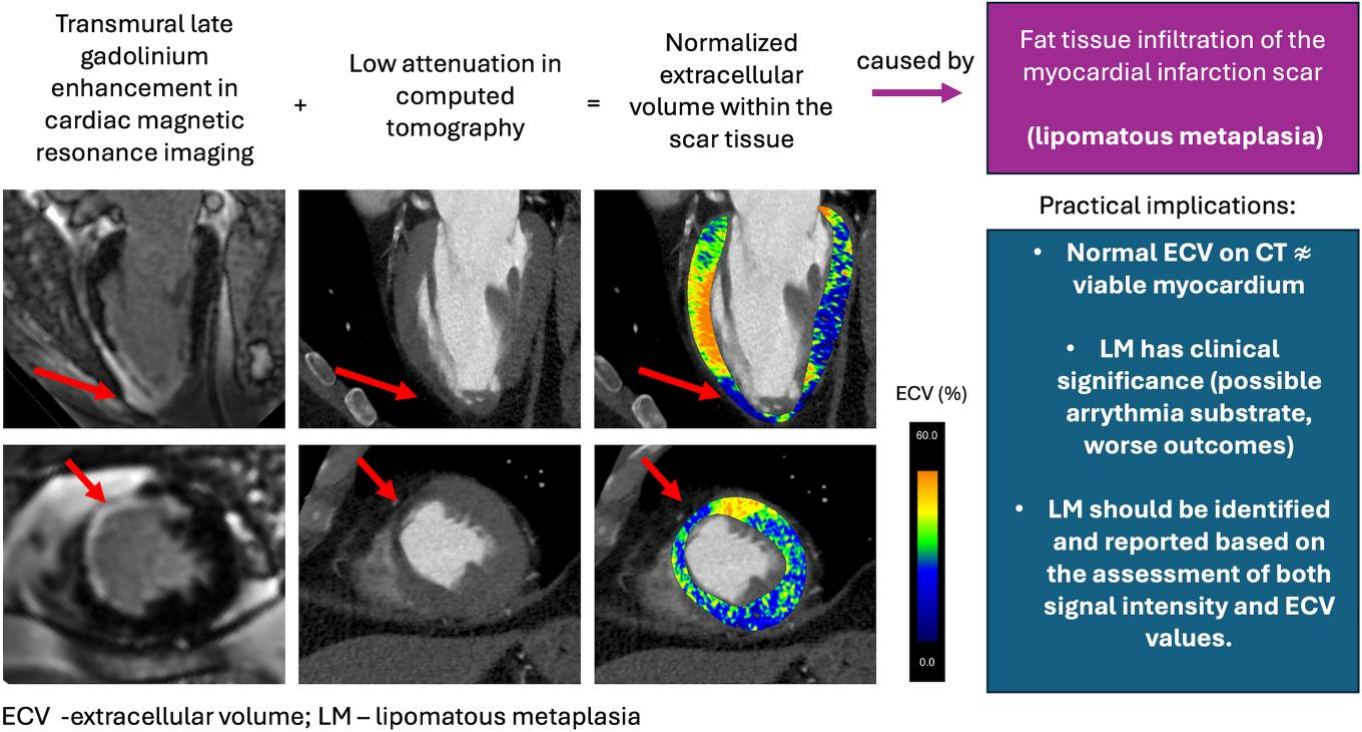


## Case presentation

**Figure 1:**
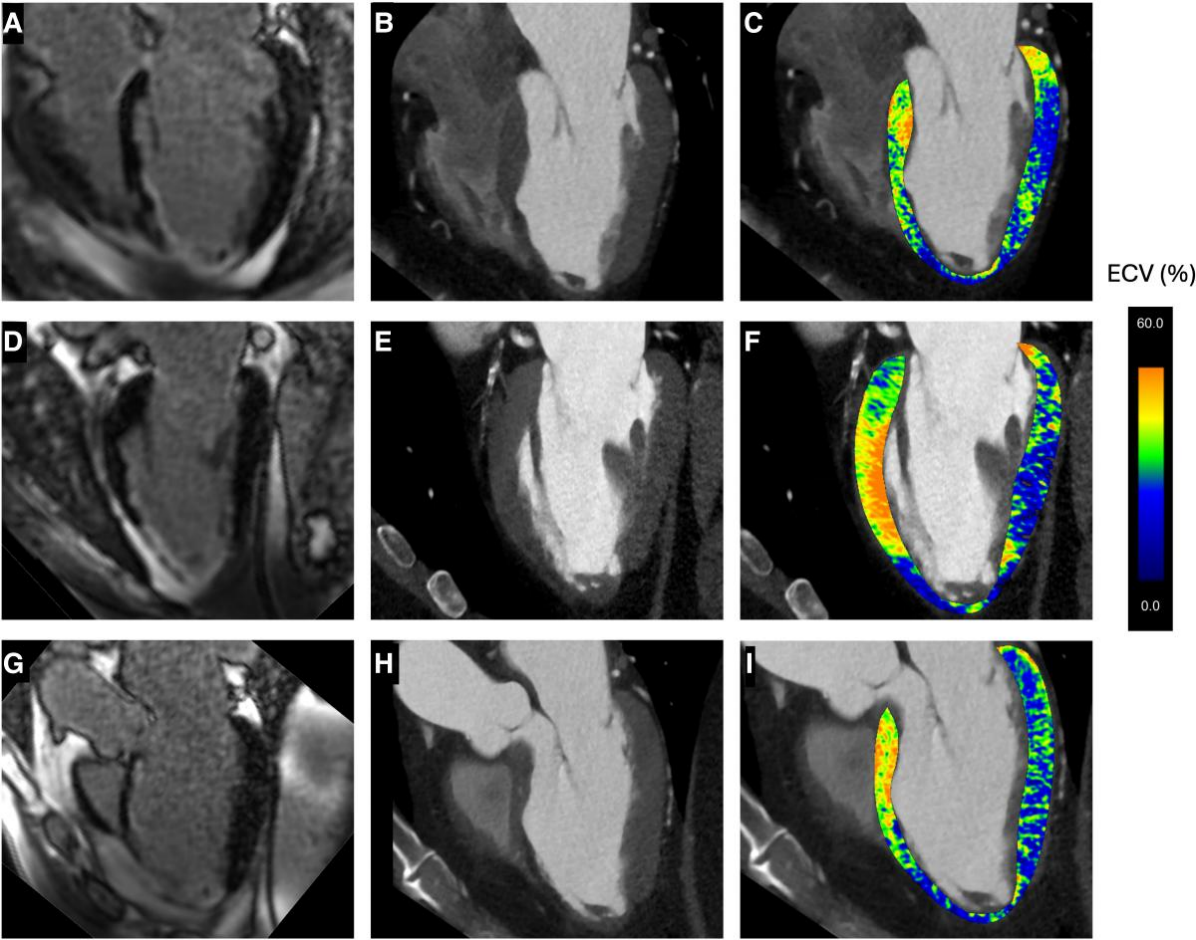
Multimodal imaging of lipomatous metaplasia (LM) – long axis planes. Panels A–I are arranged in rows corresponding to four- (A–C), two- (D–F), and three-chamber apical views (G–I) of the left ventricle. The left column (*A*, D, *G*) CMR images of late gadolinium enhancement, the middle column (*B*, E, *H*) shows corresponding CT angiography images, and the right column (*C*, F, *I*) displays colour-coded ECV values overlayed on the CT image. Abbreviations: CMR – Cardiac Magnetic Resonance, CT – Computed Tomography, ECV – Extracellular Volume.

**Figure 2:**
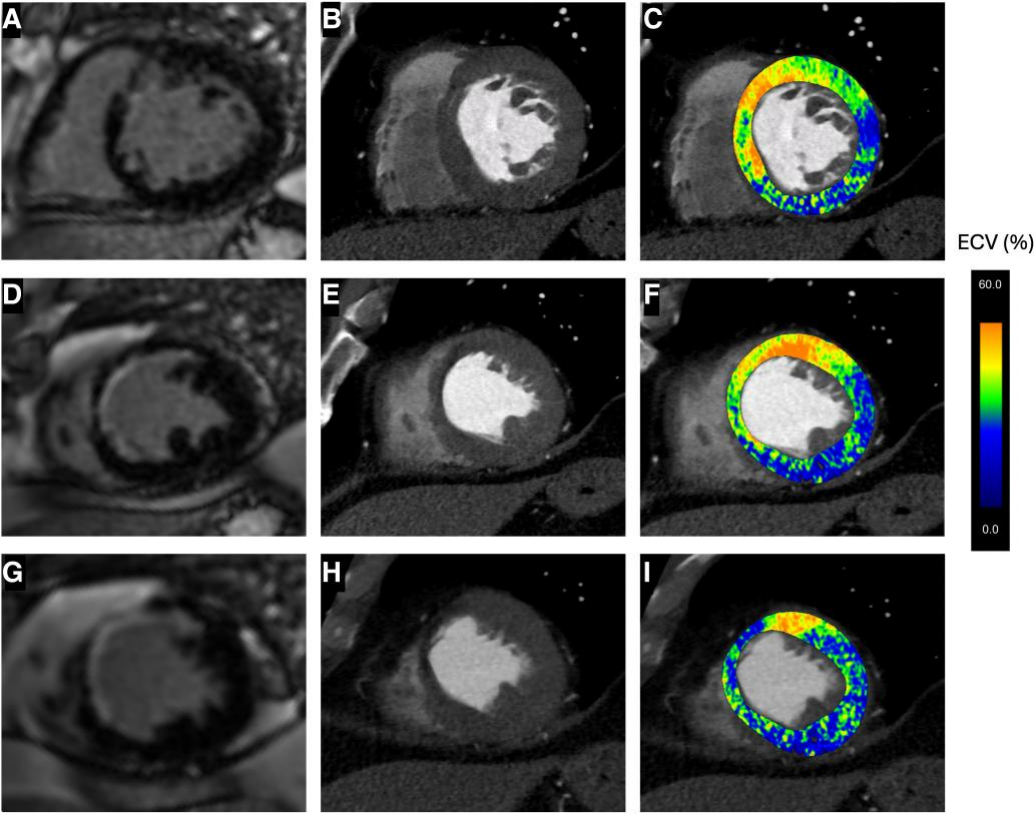
Multimodal imaging of lipomatous metaplasia (LM) – short axis planes. Panels A–I are arranged in rows corresponding to basal (*A–C*), mid (*D–F*), and apical (*G–I*) left ventricular segments. The left column (*A*, *D*, *G*) shows short-axis CMR images of late gadolinium enhancement, the middle column (*B*, *E*, *H*) shows corresponding CT images, and the right column (*C*, *F*, *I*) displays colour-coded ECV values overlayed on the CT image. Abbreviations: CMR – Cardiac Magnetic Resonance, CT – Computed Tomography, ECV – Extracellular Volume.

Cardiac magnetic resonance imaging (CMR) was performed first to determine the viability within the affected territories. The functional study identified moderately reduced left ventricular (LV) ejection fraction (LVEF) of 38% with akinesis and thinning of antero-septal segments and a minor dyskinetic apical aneurysm with accompanying small thrombus. The CMR study revealed a transmural scar in the antero-septal apical region extending to non-transmural scar in antero-septal mid-basal segments, as well as subtle sub-endocardial fibrosis in the infero-septal mid-basal segments (*Figures 1* and *2*). T1 mapping was not performed during CMR. The pharmacological stress test with regadenoson revealed mild peri-necrosis ischaemia extending beyond the areas affected by fibrosis: In the mid-anterior segments as well as in the infero-septal mid-basal segments. Further evaluation by computed tomography (CT) was recommended to assess risk and difficulty of the potential revascularization and guide the eventual interventional treatment.

**Figure 3:**
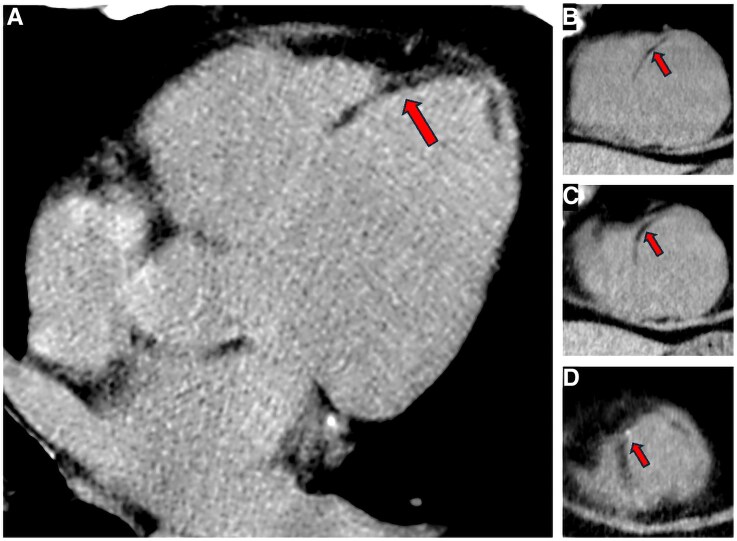
Lipomatous metaplasia visualized using dual-energy CT. The panels show the post-contrast dual-energy CT images processed for the energy threshold of 40 keV in long axis four-chamber view (*A*) as well as short axis views of the basal (*B*), mid (*C*) and apical (*D*) segments. Red arrows indicate the regions with lipomatous metaplasia. Abbreviations: CT – Computed Tomography.

Cardiac CT angiography using a dual-energy CT scanner (DE-CT) was performed, enabling a comprehensive assessment of coronary anatomy, myocardial extracellular volume (ECV), and tissue characterization. The anatomical and tissue composition results were in agreement with prior ICA and CMR findings, but the ECV analysis revealed normal ECV values in the inferior myocardial segments (where only subtle necrosis was detected by CMR), elevated ECV values (36%) in the basal and mid-anterior segments (corresponding with the presence of sub-endocardial scar in CMR), and surprisingly normal ECV values (29%) in the antero-septal apical segments that corresponded to the territory affected by transmural necrosis with thinning in CMR (*Figures 1* and *2*). This region revealed a reduced attenuation [50 ± 9 Hounsfield Units (HU)] that was more pronounced when dual energy analysis with the threshold of 40 keV was applied (*Figure 3*). This latter discordance between the presence of transmural fibrosis with normalized values of ECV was attributed to lipomatous metaplasia (LM) that formed in the myocardial scar. Secondary analysis of CMR cine imaging revealed chemical shift artefacts within the antero-septal, mid-apical segments, concordant with the presence of adipose tissue within the scar (see Supplementary material online, *Video S1*). The complexity of the CTO lesions in the LAD and PDA was assessed using the Korean CTO (KCCT) score,^1^ yielding scores of 3 (very difficult) and 2 (difficult), respectively. Considering the high CTO complexity and limited myocardial viability, a shared decision was made with the patient to defer revascularization. On follow-up at six months, the patient's condition was unchanged. He remained clinically stable with good functional capacity and well-controlled grade II angina, supporting the continued strategy of management with optimal medical therapy.

## Discussion

Lipomatous metaplasia (LM) consists of mature white adipose tissue within infarcted myocardium, though its formation remains poorly understood. One unproven hypothesis suggests it originates from the transformation of fibrocytes or multipotent stem cells into adipocytes.^2^ Areas of myocardial infarction (MI) scar display abnormal metabolism with reduced fatty acid uptake, leading to the replacement of collagen with adipocytes.^3^ This process might be predisposed by the blood lipoprotein levels as suggested by Lucke *et al*.^4^ Alternatively, T1 time shortening from methaemoglobin suggests LM may be driven by infarct zone haemorrhage.^5^

Histological studies reveal that LM can be detected in up to 84% of post-MI hearts,^6^ although its extent varies, while imaging studies report an overall incidence ranging from 22% to 68%. Furthermore, LM appears to have a progressive course, as its prevalence identified by CT increases from 0% in recent infarcts to 89% in those older than six years.^7^ The extent of LM was also shown to be positively correlated with infarct volume and reduced LV contractility.^8^ Although CMR provides significant advantages in tissue characterization, fully utilizing these capabilities may necessitate additional sequences—such as pre-contrast T1-weighted imaging or water-fat separation—which are not typically included in routine evaluations of chronic coronary artery disease and viability assessment.^9^

LM should not be considered benign, given the growing evidence linking it to older infarcts, large myocardial territories, and the development of ventricular aneurysms, all of which may contribute to adverse outcomes.^9^ The presence of LM has been associated with worse infarct healing and persistently poorer systolic function. Similarly, the presence of iron (detectable using T2*) and oedema within the infarct core are also linked to adverse remodeling.^10^ Furthermore, it influences electrical conductivity within fibrotic scar tissue, promoting the formation of scar-related ventricular tachycardia (VT) circuits, as demonstrated in an ovine model.^11^ Co-registration of CT images with electroanatomic mapping has identified LM as the most reliable CT predictor for the functional substrate in scar-related ventricular tachycardia.^12^

With high spatial resolution and 3D volumetric assessment, CT is an accurate and accessible method to detect, localize, and quantify LM. It can be visualized and quantified in both non-contrast and contrast-enhanced cardiac CT as a clearly demarcated region of lower attenuation within the LV wall,^7^ allowing the detection of areas as small as 1 mm².^9^ CT enables accurate ECV measurement, showing excellent correlation with CMR-derived values (mean difference <1%); in particular, DE-CT have demonstrated a stronger correlation with CMR-derived ECV than single-photon emission computed tomography (SPECT).^13^ Although CT-based ECV quantification is not currently considered as a viable alternative to CMR for viability assessment, it offers several advantages. CT imaging is faster, more widely available and may be more acceptable in patients with claustrophobia or those with cardiac devices that might not be MR-compatible.^14^ CT can also provide high-resolution, 3-dimensional volumetric ECV measurement with whole-heart acquisition, minimizing motion artefact. A key advantage is the ability to co-register and visualize zones of altered ECV with the coronary anatomy.

To our knowledge, this is the first report to underscore the normalization of CT-derived ECV values in chronic myocardial scars containing LM. This phenomenon occurs because adipose infiltration displaces the interstitial space, minimizing extracellular expansion and thus masking the underlying scar.^6^ A similar biasing effect on T1-based ECV maps was previously reported in CMR.^15^ Recognizing this pitfall is critical, as deceptively normal ECV values can lead to misclassification of myocardial viability and affect management decisions.

## Conclusions

Our case demonstrates that lipomatous metaplasia in a chronic infarct can normalize CT-derived ECV values, falsely suggesting myocardial viability. This finding should alert imaging specialists that, in the setting of chronic infarction, normal ECV may conceal advanced myocardial tissue injury. Further studies are needed to guide treatment based on the presence of LM and to understand how its formation may be affected by optimal medical therapies, including lipid-lowering treatments.

## Lead author biography


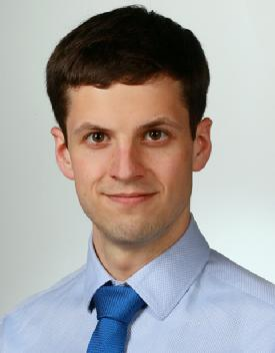


Konrad Pieszko MD, PhD works in the Cardiology Clinic of the University of Zielona Góra, Poland, and currently participates in advanced cardiac imaging fellowship at Sant Pau Research Institute–Hospital de la Santa Creu i Sant Pau, Barcelona. His research integrates artificial intelligence with multimodal cardiac imaging including CT, PET/SPECT, CMR and echocardiography to improve risk stratification.

## Supplementary Material

ytaf612_Supplementary_Data

## Data Availability

The raw imaging data underlying this case report are not publicly available to protect patient privacy. De-identified images may be available from the corresponding author upon reasonable request and with appropriate institutional approval.
