# Supplementary material for: Lipomatous metaplasia in cardiac CT: when ‘normal’ extracellular volume does not indicate myocardial viability - a case report
Source: Eur Heart J Case Rep. 2025 Dec 1;9(12):ytaf612. doi: 10.1093/ehjcr/ytaf612 (PMC12693519; doi:10.1093/ehjcr/ytaf612)
Supplement: ytaf612_Supplementary_Data [file ytaf612_supplementary_data.zip › Supplemental File Legend.docx]

**Supplemental File Legend**


1. **Supplemental Video 1.** Cine imaging in 3-chamber view revealing chemical shift artifacts within the antero-septal, mid-apical segments, concordant with the presence of lipomatous metaplasia.
